# Experiential seizures related to the hippocampal-parahippocampal spatial representation system

**DOI:** 10.1016/j.ebr.2020.100386

**Published:** 2020-08-31

**Authors:** Eline Revdal, Vibeke Arntsen, Thanh Pierre Doan, Marte Kvello-Alme, Kjell Arne Kvistad, Geir Bråthen, Eylert Brodtkorb

**Affiliations:** a Department of Neurology and Clinical Neurophysiology, St.Olav University Hospital, Trondheim, Norway; b Department of Neuromedicine and Movement Science, Norwegian University of Science and Technology, Trondheim, Norway; c Kavli Institute for Systems Neuroscience, Center for Computational Neuroscience, Egil and Pauline Braathen and Fred Kavli Center for Cortical Microcircuits, Norwegian University of Science and Technology, Trondheim, Norway; d Department of Psychiatry, Nord-Trøndelag Hospital Trust, Levanger Hospital, Levanger, Norway; e Department of Radiology, St.Olav University Hospital, Trondheim, Norway

**Keywords:** Visual hallucinations, Experiential seizures, Focal aware seizures, Hippocampal-parahippocampal system, Parahippocampal cortex

## Abstract

Ictal visual hallucinations may have occipital as well as temporal lobe origin. We report a patient with clustering of focal aware seizures with visual hallucinations. Ictal EEG findings and seizure semiology with alternating contralateral elementary visual phenomena and non-lateralizing experiential hallucinations (visual scenes, memory flashbacks, spatial distortion) corresponded to a lesion in the posterior part of the right parahippocampal gyrus. This area is part of the hippocampal-parahippocampal system for mapping allocentric space. Within this system, the parahippocampal cortex encodes information about visual environmental scenes in concert with functionally defined neurons relevant for episodic memory and spatial cognitive processes (place, grid, border and head direction cells, as well as neurons tracking the passage of time). These functions are tightly linked to visual exploration.

We suggest that the hippocampal-parahippocampal spatial navigation system is a crucial part of the networks responsible for the semiology of experiential seizures with complex visual hallucinations and elements of recall.

## Highlights

• Ictal visual hallucinations may have occipital or temporal lobe origin.
• In temporal lobe epilepsy, they may be experiential and non-lateralized.
• In the present patient, such seizures were related to a parahippocampal lesion.
• This area overlaps with the spatial representation systems of the brain.
• A more precise outline of the substrate for experiential seizures is suggested.

## 1 Introduction

Hallucinations in people with epilepsy can be divided as ictal, postictal and interictal according to their relationship to seizure events [1]. Ictal symptoms are determined by the function of the seizure-generating area in the brain, i.e. the area of ictal onset and propagation in synaptically connected network areas. Ictal visual hallucinations may have occipital as well as temporal lobe origin. In occipital lobe epilepsy, they consist of elementary sensory symptoms restricted to one visual field. In temporal lobe epilepsy, they may be elementary and lateralized, as well as complex and experiential without confinement to any visual field. Experiential seizures may encompass vivid memory-like hallucinations, such as scenes from the past [[2], [3], [4], [5], [6], [7], [8]].

We present a case of focal epilepsy and prolonged bouts of mixed elementary and complex visual hallucinations due to a lesion in the right parahippocampal cortex. The neuronal correlates of spatial cognition and episodic memory in relation to visual representations are discussed with respect to the hippocampal-parahippocampal systems for mapping allocentric space [9]. The present case study illustrates the value of systems neuroscience to examine how psychiatric and neurological symptoms can manifest through comparable pathophysiological mechanisms within overlapping neural networks [10].

## 2 Case history

### 2.1 Background

**Fig. 1:**
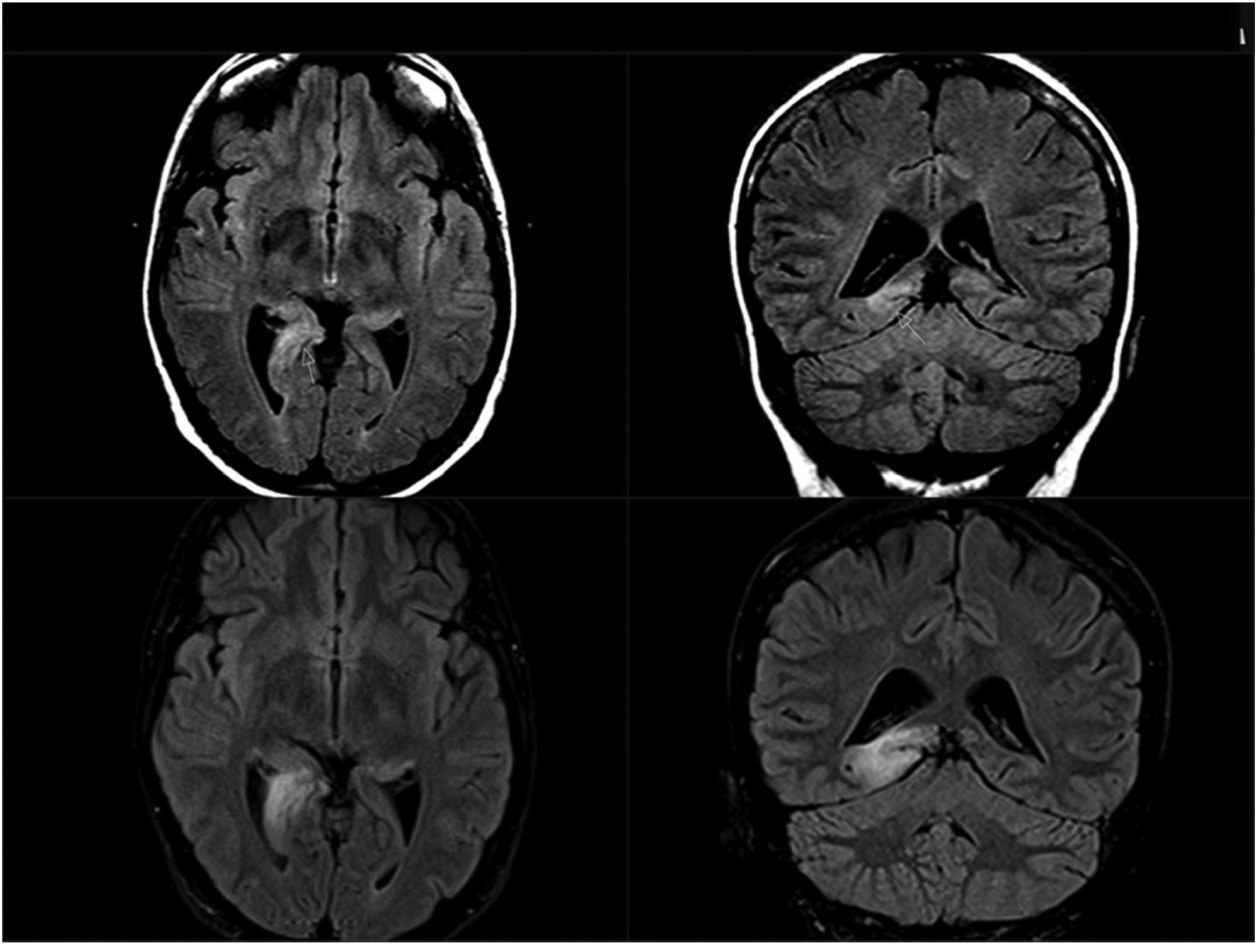
MRI findings. FLAIR images (axial plane, left; coronal plane, right). Upper: Prior to serial seizures with complex hallucinations. Lesion in the posterior part of the right parahippocampal gyrus and in the hippocampal tail. Lower: During serial seizures. Substantial volume increase of the lesion with extension toward the occipital cortex.

Since her mid-twenties, this right-handed woman presented focal to bilateral tonic–clonic seizures (FTCS) during sleep. EEG showed right-sided temporal slow activity, and MRI demonstrated an abnormality in the posterior part of her right parahippocampal gyrus initially interpreted as gliosis (Fig. 1, upper part).

She was treated with lamotrigine (LTG), but still had occasional nocturnal FTCS. She became pregnant at age 31. Adjustments of LTG doses according to declining serum concentrations failed to maintain pre-pregnancy levels. In the last months of gestation, she reported episodes of déjà vu followed by visual phenomena in her left visual field. These included flashing lights as well as seeing a mirror reflection of herself (autoscopy).

### 2.2 Ictal hallucinations

After another nocturnal FTCS in gestational week 36, she became confused and agitated. She screamed, ran around, and tried to jump off her balcony. A psychosis was suspected, and she was acutely admitted to psychiatric hospital. These symptoms quickly resolved. She had amnesia for the entire episode. Later, she reported recurrent flashing lights evolving to vivid complex visual hallucinations perceived as revivals of previous dreams and experiences. Interictal EEG showed epileptiform discharges in her right posterior temporal area.

Eight days after the acute event, MRI showed a substantial volume increase of the lesion in her right posterior temporal lobe toward the corresponding posterior horn of the lateral ventricle and retrosplenial cortex. The changes were interpreted as cytotoxic oedema and included the hippocampus as well as the parahippocampal cortex (Fig. 1, lower part).

After delivery by caesarean section, clinical examination revealed a complete left-sided homonymous hemianopia. Frequent series of episodic visual hallucinations continued. The various semiological features are reported in Table 1. The symptoms were recognized as imaginary, precluding true psychosis. Interictally, she reported an odd feeling of spatial disorientation.

**Table 1:** Reported visual perceptions during focal aware serial seizures.

Repetitive, stereotypical and elementary visual hallucinations in the left visual field:
- Bright flickering light, partly as intense sunshine
- Mirror reflection of myself


Diffusely distributed complex, moving and non-stereotypical visual hallucinations and illusions in the entire visual field, sometimes with left-sided predominance:
- Difficulty reading and writing, letters change form and position, transform to strange signs, resembling hieroglyphics – cannot control where my pen hits
- The room turning smaller and darker and then larger and brighter
- Familiar sceneries of landscapes, trees, running water, rivers
- Beaches and cottages/boathouses by the sea, reminiscent of previously visited holiday locations
- People with shopping bags passing at the local supermarket
- Distortion of people's faces turning purple; blood
- Hair growing in people's faces, covering only parts of the face or the entire face, as if people turn into werewolves
- Moving purple spots and stains of oil – colourful – to the left – also all over the room – difficult to explain
- Crawling bugs and spiders

**Fig. 2:**
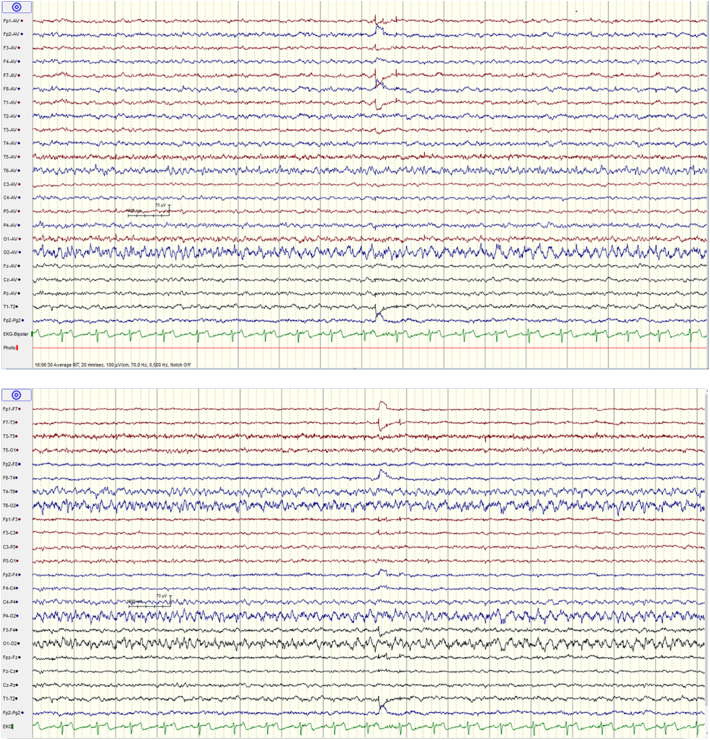
Stereotypical ictal EEG activity associated with non-lateralizing visual hallucinations with spatial distortion during long-term monitoring (time 04.06.30 PM, see Table 2). Montage: average, upper; bipolar longitudinal, lower. Electrographic seizures begin with slow 3-Hz delta activity in the right occipital and posterior temporal region (leads O2 and T6), evolve to spiky rhythmic 8-Hz activity; midway, the frequency declines to 4-Hz theta activity.

Long-term video-scalp EEG was performed during episodic visual hallucinations and demonstrated corresponding ictal activity in the right temporo-occipital area (Fig. 2). Subclinical ictal activity was recorded during sleep. In Table 2, ictal experiences during long-term EEG are reported *verbatim* as written by the patient, seemingly hampered by visual and spatial disturbances. During the first recorded cluster, four clinical seizures occurred within 16 min.

**Table 2:** Ictal EEG activity and clinical symptoms recorded during long-term monitoring.

Scalp EEG	Digital time PM	Time PM, recorded by patient	Ictal symptoms *verbatim* reported in writing
Ictal activity	03:48:41–03:49:54 1 m, 11 s	03:50	*Reading and writing*: Difficulties; the letters rearrange.
EEG not saved		3:55	*Writing SMS*: Difficult, letters appear misplaced; they look like different signs.
EEG not saved		04:02	*Reading book*: Letters change place; move – understand the word, but misspelt.
Ictal activity	04:05:58–04:07:06 1 m, 8 s	04:06	Visions, predominantly to the left, but then all over the table in front of me/to the side/the room looks different – also some flashings, just lasting ½ min.
Ictal activity	04:21:59–04:22:39 38 s	04:20	*Awakening from nap*: Very strong sunshine up to the left. Water, beach, boathouses, beautiful places. Everything within 1 min. Difficult to read what I am writing now.
Ictal activity	05:40:36–17:41:39 1 m, 3 s		Seizure not reported
Ictal activity	07:14:13–19:15:32 1 m, 19 s	07:15	Visions, seeing myself in bed – up to the left – boathouses, less than 1 min. *Watching TV*: Lots of flashings up to the left; first visions on the screen; the notepaper looks different. Visions on the wall lasting about 1 min. My sight is very poor; cannot read what I am writing.
No ictal activity		07:35	*Watching TV/looking out of the window*: Poor vision, as if I am not wearing lenses, but they are both in place.
No ictal activity		08:30	Lots of hallucinations in my entire visual field; scenery etc.; mixed with what is actually in the room. I know what is real, as I recall how it looked like a couple of minutes ago. The room changes – gets smaller and darker, then bigger and brighter, but I know what is real. The hallucinations last less than 1 min, but come and go with short intervals. Poor vision makes me unable to control my spelling.
Ictal activity	10:59:43–11:01:14 1 m, 31 s	11:00	*Surfing on the mobile*: Hallucinations appear as soon as I start reading. See many things on the walls for about 30 s. My eyesight gets worse. Difficult to read, even large font.

SMS, short message service (cell phone).

### 2.3 Outcome

Add-on valproate controlled the seizures for several weeks. Another MRI after seven weeks showed a complete return to pre-pregnancy state, suggesting that the changes represented post-ictal phenomena (Fig. 1). Clinically, the visual field defect had also resolved.

Later, focal aware seizures recurred. This time, the initial phenomena consisted of déjà vécu with an idea that looking at a random object induced seizures. Visual symptoms were limited to simple left-sided phosphenes with a perception of the surroundings turning unique and spectacular, along with anxiety, palpitations and abdominal discomfort. She developed drug-resistant epilepsy, and she was eventually referred for epilepsy surgery assessment three years later. A depth electrode EEG recording (now in the absence of ictal scalp EEG activity) demonstrated ictal onset related to the lesion in her right parahippocampal gyrus. A lesionectomy was performed. Histological examination revealed a ganglioglioma grade 1. Following the surgery, she was left with aware seizures with an initial odour followed by déjà vu sensations. FTCS and complex hallucinations have not recurred.

Ethical approval other than collection of written informed consent was not required by the Regional Committee for Ethics in Research.

## 3 Discussion

The patient developed an enduring state of relapsing and remitting ictal symptoms lasting several days, approximating focal status/“aura continua” [11,12]. The excessive focal seizures appeared to cause a visual field deficit corresponding to an increase of MRI FLAIR signal in the vicinity of the lesion, likely caused by sustained cerebral hypoperfusion [13,14]. Presumably, these transitory changes enhanced ictal activity and propagation causing further neuronal damage, which may have contributed to subsequent drug-resistance [15].

Ictal visual symptoms may present as elementary hallucinations (seeing bright spots or simple geometrical figures) typically originating in the primary visual cortex, whereas complex hallucinations, such as seeing whole scenes, have been suggested to involve the visual association areas in the temporal/parietal lobes [16]. A series of surgically treated patients with ictal visual symptoms clearly demonstrated that elementary hallucinations can also occur with temporal lobe onset (anteromedially and posteriorly), similarly to the present patient, while complex hallucinations never occurred in distinct occipital lobe onset seizures [17]. In that study, elementary visual hallucinations were confined to the contralateral visual field, whereas a lateralization of complex hallucinations was not reported. Still, in all cases, ictal EEG activity was localized to a lesion demonstrated by MRI or to its neighbouring regions [17]. Elliott and Shorvon claim that complex hallucinatory experiences in epilepsy cannot be well localized, and the more elementary they are, the more localized they tend to be [6]. Noteworthy, simple autoscopic mirror images are likely to be lateralized [18].

In the present patient, non-lateralizing, abundant and non-stereotypical experiential phenomena were associated with ictal temporo-occipital scalp EEG activity corresponding to a parahippocampal lesion. This constellation calls for further explanation. The lesion was located within the posterior portion of the medial temporal lobe corresponding to the parahippocampal cortex where both functional MRI signal and single unit recordings have revealed neurons encoding visual scenes selectively [19,20]. This particular area is part of the hippocampal-parahippocampal system for mapping allocentric space. It contains several functionally specific neurons associated with spatial cognition and episodic memory, such as place, grid, border and head direction cells [9,21], as well as neurons tracking the passage of time [22,23]. In rodents, these functionally defined neuronal types are phylogenetically preserved across mammals and have also been reported in primates, including humans [[24], [25], [26]]. Although associated with visual phenomena, this spatial navigation system does not relate to any visual field lateralization. Based on single hippocampal neuron recordings during episodic memory recall in humans [27], it might be speculated whether activation of subsets of these specific cell populations could elicit experiential phenomena related to recollection of previously experienced scenarios and situations (landscapes, moving elements, faces) in the form of a “mental diplopia”. Consistent with this hypothesis, it has been reported that electric stimulation of the parahippocampal place-selective area elicited various topographical visual hallucinations with qualities of déjà vu [28]. Conceivably, these functionally defined neurons act in concert with synaptically connected areas within various “visual streams” and epileptic pathways. A complex functional connectivity based on multiple and bidirectional epileptic propagation within these subcortical networks may confuse the relationship between the epileptogenic and symptomatogenic zones and thus explain the abundant and variable semiology [29]. Advanced stereo-EEG recordings during experiential seizures might further map the responsible neuronal networks [28,30].

These recent findings now add to the understanding of experiential phenomena in people with temporal lobe epilepsy, so wonderfully described and discussed in detail by Penfield [2,3] and Gloor [4] several decades ago. The medial temporal lobe navigation system seems to shape our visual experience [31]. We believe that the present example illustrates the relevance of a more precise delineation of the principal symptomatogenic zones and networks responsible for this seizure type. A lateralizing value of experiential seizures is dubious, as these phenomena have been recorded or elicited from both hemispheres [[2], [3], [4], [5],^8^,^28^], although intuitively, visuo-spatial phenomena may be thought to predominate from the non-dominant hemisphere [3].

## 4 Concluding remarks

The electroclinical features in our patient alternated between contralateral elementary and non-lateralized complex hallucinations in the form of visual scenes due to a lesion in the right parahippocampal cortex. We hypothesize that activation of the hippocampal-parahippocampal spatial navigation systems causes experiential hallucinations by interaction with visuospatial networks. Specific neuronal populations associated with spatial cognition and episodic memory may be responsible for the symptomatology, conceivably together with propagation to pathways with neocortical cognitive and sensory functions. These networks may be part of the anatomical substrate for experiential seizures.
